# Bortezomib and dexamethasone for multiple myeloma: higher AST and LDH levels associated with a worse prognosis on overall survival

**DOI:** 10.1186/1471-2407-14-462

**Published:** 2014-06-21

**Authors:** Takayoshi Kiba, Takuo Ito, Toshihisa Nakashima, Yoshiko Okikawa, Miki Kido, Akiko Kimura, Keita Kameda, Fumiaki Miyamae, Suzuko Tanaka, Misao Atsumi, Yoko Sumitani, Yoshimi Shitakubo, Hiromasa Niimi

**Affiliations:** 1 Division of Modern Medical Technology, Institute for Clinical Research, National Hospital Organization Kure Medical Center and Chugoku Cancer Center, 3-1, Aoyama-cho, Kure-shi, Hiroshima 737-0023, Japan; 2 Department of Hematology and Oncology, National Hospital Organization Kure Medical Center and Chugoku Cancer Center, 3-1, Aoyama-cho, Kure-shi, Hiroshima 737-0023, Japan; 3 Department of Pharmacy, National Hospital Organization Kure Medical Center and Chugoku Cancer Center, 3-1, Aoyama-cho, Kure-shi, Hiroshima 737-0023, Japan; 4 Clinical Trial Management Office, National Hospital Organization Kure Medical Center and Chugoku Cancer Center, 3-1, Aoyama-cho, Kure-shi, Hiroshima 737-0023, Japan

**Keywords:** Bortezomib, Multiple myeloma, Prognosis

## Abstract

**Background:**

Bortezomib offers a novel approach to the treatment of multiple myeloma producing rapid control. The aim of this study was to investigate the outcomes of bortezomib and dexamethasone-treated patients with multiple myeloma.

**Methods:**

We conducted a retrospective study of 44 consecutively-treated multiple myeloma patients with bortezomib (1.3 mg/m^2^ on days 1, 4, 8, and 11 of a 21-day cycle or 1.3 mg/m^2^ intravenously 1, 8, 15, and 22 of every 35-day cycle) and dexamethasone.

**Results:**

The median time to progression, progression free survival time, and overall survival time in the treatment groups was 14.9, 14.9, and 38.3 months, respectively. The present study also suggests the possibility that the prognosis of patients with high levels of AST and LDH might be worse.

**Conclusions:**

Our results indicate that the treatment of multiple myeloma with bortezomib and dexamethasone is feasible.

## Background

Multiple myeloma is a plasma cell neoplasm that accounts for approximately 10% of all hematologic malignancies [1]. A diagnosis of myeloma requires the presence of 10% or more clonal plasma cells on bone marrow examination and/or a biopsy-proven plasmacytoma, as well as evidence of end-organ damage (i.e., hypercalcemia, renal insufficiency, anemia, or bone lesions) that is attributable to the underlying plasma cell disorder [2]. The treatment of multiple myeloma (MM) is evolving rapidly [3]. There are at least five active classes of treatment: alkylators (e.g., melphalan and cyclophosphamide), corticosteroids (e.g., prednisone and dexamethasone), proteasome inhibitors (e.g., bortezomib and carfilzomib), immunomodulatory drugs (e.g., thalidomide and lenalidomide), and anthracyclines (e.g., doxorubicin and liposomal doxorubicin). Melphalan-prednisone (MP) was introduced for the treatment of MM in the late 1960s. In the subsequent 30 years, treatment improvements remained stagnant, since more complex chemotherapy combinations, such as vincristine, doxorubicin, and dexamethasone (VAD), or with the addition of BCNU (VBAD) or melphalan and cyclophosphamide (VCMP), only led to small increases in the overall response rate but without differences in survival, as assessed in a large meta-analysis that included over 6,000 patients. The next step forward was the use of high-dose melphalan followed by stem cell support (autologous stem cell transplant - ASCT) for young myeloma patients, which resulted in a significant improvement in progression-free survival and overall survival. However, for elderly patients, MP remained the standard of care. From the year 2000, a revolution in the treatment armamentarium of MM has emerged with the availability of new agents with a singular mechanism of action such as thalidomide and lenalidomide, both immunomodulatory drugs, and the proteasome inhibitor bortezomib [4]. A plethora of doublet, triplet, and quadruplet combinations have been studied for the treatment of newly diagnosed myeloma. Although randomized trials have been conducted comparing older regimens such as MP with newer regimens containing drugs such as thalidomide, lenalidomide, or bortezomib, there are few if any randomized trials that have compared modern combinations with each other. Even in the few trials that have done so, definitive overall survival or patient-reported quality-of-life differences have not been demonstrated. Therefore, there is marked heterogeneity in how newly diagnosed patients with myeloma are treated around the world. The choice of initial therapy is often dictated by availability of drugs, age and comorbidities of the patient, and assessment of prognosis and disease aggressiveness [3].

In the present study, we retrospectively analyzed the efficacy and safety of bortezomib and dexamethasone in the treatment of patients with MM treated at the National Hospital Organization Kure Medical Center and Chugoku Cancer Center. The prognostic factor for survival in MM patients receiving bortezomib was also retrospectively investigated in this study using Cox regression analysis. In addition, the current status of studies aimed at understanding these results was also reviewed.

## Method

### Ethics statement

Only demographic data of patients were stored in the hospital database to enable retrieval of files manually based on patient codes. Charts and discharge summaries were perused. The study was investigated in accordance with the ethical principles stated in the most recent version of the Declaration of Helsinki or the applicable guidelines on epidemiological studies issued by the Ministry of Health, Labor and Welfare and the Ministry of Education, Culture, Sports, Science and Technology, Japan, whichever represented the greater protection to the individual ( http://www.mhlw.go.jp/). All data were anonymously analysed without individual patient consent due to the retrospective nature of the study. In addition, the National Hospital Organization Kure Medical Center and Chugoku Cancer Center Institutional Review Board Ethics Committee waived the need for individual informed consent and approved the study (Approval Number G25-03, date 10/29/13).

### Patients

We conducted a retrospective study of 44 patients treated with bortezomib and dexamethasone therapy between March 2008 and October 2012. All patients who had received at least one cycle of treatment that included bortezomib were analyzed in this retrospective study. The diagnosis of MM was confirmed using the International Myeloma Working Group (IMWG) criteria [5]. In the present study, we did not collect data on the patients diagnosed with plasma cell leukemia. Table 1 shows the characteristics of the 44 patients just before the bortezomib therapy. The clinical stage was determined by the Durie-Salmon classification and the International Staging System (ISS) [6,7]. The median age was 71 years old (49–86 years old), with 19 males and 25 females. Most (72.7%) had IgG or IgA myeloma. Fifteen (34.1%) received autologous stem-cell transplantation.

**Table 1:** Patient characteristics (n = 44)

**Median age, years (range)**	**71 (49–86)**
Gender Male/female	19/25
Performance status	
0	24 (54.5)
1	14 (31.8)
2	4 (9.1)
≥3	2 (4.5)
Type of M protein *n* (%)	
IgG	22 (50.0)
IgA	10 (22.7)
IgD	1 (2.3)
BJP	10 (22.7)
No secreted	1 (2.3)
Durie-Salmon stage *n* (%)	
I	4 (9.1)
II	6 (13.6)
III	34 (77.3)
ISS stage *n* (%)	
1	10 (22.7)
2	11 (25.0)
3	23 (52.3)
No. genetic abnormalities of 13q deletion *n* (%)	
Yes	6 (13.6)
No	38 (86.4)
No. of stem cell transplantation *n* (%)	
Yes	15 (34.1)
No	29 (65.9)
No. of prior treatment regimens* *n* (%) 09 (20.5)	
1	16 (36.4)
2	7 (15.9)
≥3	12 (27.3)
Type of prior treatment regimens *n* (%)	
VAD	22 (25.9)
MP	19 (22.4)
HDD	10 (11.8)
Thalidomide	9 (10.6)
Stem cell transplantation	5 (5.9)
CP	5 (5.9)
ROAD	4 (4.7)
Cyclophosphamide alone	3 (3.5)
DEX pulse	2 (2.4)
MD	2 (2.4)
INF α-MP	2 (2.4)
CAD	1 (1.2)
RD	1 (1.2)

CP: cyclophosphamide, prednisolone; CAD: cyclophosphamide, adriamycin, dexamethasone; DEX pulse: dexamethasone pulse therapy; MD: melphalan, dexamethasone; MP: melphalan, prednisolone; HDD: high dose dexamethasone; INF α: Interferon Alpha, RD: lenalidomide, dexamethasone; ROAD: MCNU, vincristine, melphalan, dexamethasone; VAD: vincristine, adriamycin, dexamethasone.

*A regimen was defined as a single drug or combination therapy. Front-line therapy could be composed of more than one regimen.

### Treatment

Forty-four patients were treated with bortezomib alone (1.3 mg/m^2^ intravenously 1, 4, 8, and 11 of every 21-day cycle or 1.3 mg/m^2^ intravenously 1, 8, 15, and 22 of every35-day cycle) in combination with dexamethasone. All patients received 8 or 16 mg of dexamethasone on the day of and the day after each of bortezomib. In cases of grade 3/4 hematological toxicity, the next chemotherapy schedule was delayed until there was a sufficient recovery of neutrophils or platelets. The dose of bortezomib would also be reduced according to the package insert (from 1.3 mg/m^2^to 1.0 mg/m^2^, from 1.0 mg/m^2^ to 0.7 mg/m^2^, from 0.7 mg/m^2^ to stopping dosage, respectively) in the subsequent cycles. In cases of grade 1/2 neuropathic pain or peripheral neuropathy, the dose of bortezomib would also be reduced according to the package insert (from 1.3 mg/m^2^ to 1.0 mg/m^2^, from 1.0 mg/m^2^ to 0.7 mg/m^2^, respectively), while in cases of grade 3/4, the next chemotherapy schedule was delayed until there was a sufficient recovery in terms of these side effects, and the dose would also be reduced according to the package insert (to 1.0 mg/m^2^, once a week). The median duration of follow-up was 17.5 months (range 0.7-58.3 months) and the median number of treatment cycles was 3 (range 1–14). Forty patients discontinued treatment because of complete response (CR) with autologous stem-cell transplantation (5 cases: 11.4%), CR without stem-cell transplantation (1 case: 2.5%), progressive disease (PD) (27 cases: 67.5%), toxicity (1 case 2.5%), and other reasons (6 cases: 15.0%) (Table 2). Of the 27 patients who discontinued bortezomib with PD, 20 (74.1%) received conventional chemotherapy (CED, EPOCH, MP, MPT, MCNU-VMP, RD, ROAD, VAD, cyclophosphamide alone, DEX pulse, thalidomide, lenalidomide, and zoledronic acid), and 7 of 20 (35.0%), who had the above chemotherapy, received stem-cell transplantation, and 7 received no additional therapy. Also, 2 of 27 patients (7.4%) patients received radiotherapy (Table 2).

**Table 2:** Reasons for discontinuation of treatment with bortezomib and dexamethasone (n = 40) and numbers of patients, which had conventional therapy in patients who discontinued bortezomib and dexamethasone with PD (n = 27)

1) No. of patients who discontinued therapy *n* (%)	40 (100)
- CR with autologous stem cell transplantation	5 (12.5)
- CR without stem cell transplantation	1 (2.5)
- PD	27 (67.5)
- toxicity	1 (2.5)
- other reasons	6 (15.0)
2) No. of patients who had conventional chemotherapy in 27 patients who discontinued bortezomib and dexamethasone with PD *n* (%)	27 (100)
- conventional chemotherapy with autologous stem cell transplantation	6 (22.2)
- conventional chemotherapy with autologous stem cell transplantation and radiotherapy	1 (3.7)
- conventional chemotherapy with radiotherapy	1 (3.7)
- conventional chemotherapy alone	12 (44.4)
- no additional therapy	7 (25.9)

CR: complete response; PD: progressive disease.

### Assessments

Progression-free survival was defined as the time from the initial administration of bortezomib to the identification date of progressive disease (PD) or death. Time to disease progression was defined as the time from the initial administration of bortezomib to disease progression or to the initiation of other therapy. Overall survival was defined as the time from the initial administration of bortezomib to death of any cause. Responses were assessed according to the IMWG uniform response criteria [8]. Briefly, a CR was defined by the absence of monoclonal immunoglobulin (M protein) in serum and urine, as confirmed by immunofixation and the disappearance of any soft tissue plasmacytomas and less than 5% plasma cells in bone marrow. PD was defined by any of the following: an increase of M protein in serum (the absolute increase must be 0.5 g/dl) or urine (the absolute increase must be 200 mg per 24 h) or more than 25%, an increase in bone marrow plasma cells (the absolute % must be more than 10%), new or increased bone lesions or plasmacytomas, or new hypercalcemia. Adverse events were assessed and graded according to National Cancer Institute Common Terminology Criteria for Adverse Events version 4.0 ( http://evs.nci.nih.gov/ftp1/CTCAE/CTCAE_4.03_2010-06-14_QuickReference_8.5x11.pdf).

### Statistical analysis

Progression-free survival, time to disease progression, and overall survival were analyzed with Kaplan-Meier methods. To identify the prognostic factors independently associated with overall survival and progression-free survival, and to estimate the hazard ratios, the Cox proportional hazards model was applied. All statistical analyses were performed using SPSS version 19.0 statistical software. A value of 0.05 indicated statistical significance.

## Results

### Patient characteristics

Nineteen males and 25 females with a median age of 71 years (range 49–86 years) were treated consecutively with bortezomib. Table 1 shows baseline patient characteristics and a summary for MM patients. Patients received a median of 1 therapy (range 0–8) prior to bortezomib retreatment and a median of 3 cycles of bortezomib (range 1–14) as retreatment; 86.4% received 1–6 cycles.

### Efficacy

**Figure 1:**
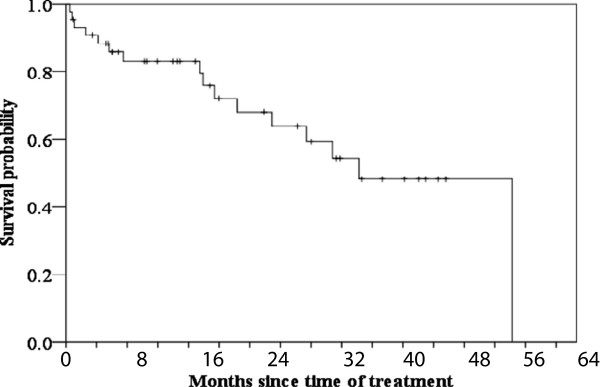
Overall survival curves in bortezomib treated MM patients.

**Figure 2:**
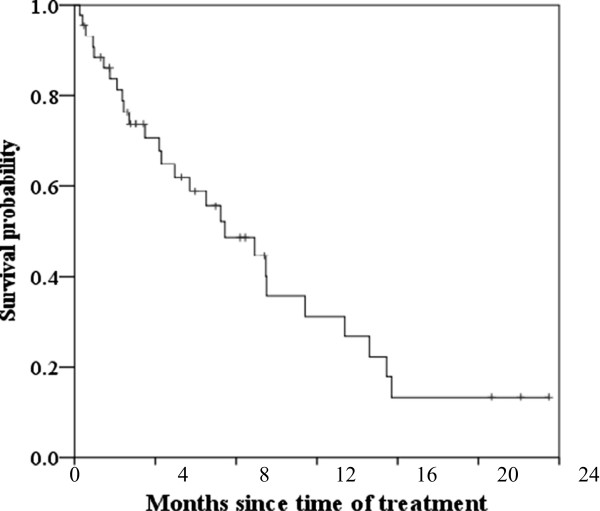
Progression-free survival curves in bortezomib treated MM patients.

A total of 627 instances of chemotherapy were performed with a median of 11.5 instance (range 3–56). The median dose for these patients was 11.86 mg/m^2^. The median overall survival time, progression-free survival, and time to progression of MM was 38.3 months (95% CI: 29.0-47.5 months), 14.9 months (95% CI: 7.6-22.2 months), 14.9 months (95% CI: 7.6-22.2 months), respectively (Figures 1 and 2). Associations between overall survival and progression-free survival and patient characteristics (age, gender, ECOG performance status, Durie-Salmon stage, International Staging System (ISS) stage, type of M protein, genetic abnormalities of 13q deletion, stem cell transplantation, hematologic and biochemical measurements, β2-microglobulin) were analyzed. In the present study, we did not detected any high risk marker including del(17p) and t(4;14) in the treated patients. The t(4;14) translocation is undetectable by conventional cytogenetics. In general, t(4;14) translocation is detected by interphase FISH. FISH testing for MM is indicated in individuals who have been diagnosed with MM based on bone-marrow cells, which have the characteristics of morphology, cytochemical staining, and immunophenotype. Univariate Cox regression analyses to determine prognostic factors associated with overall survival revealed 13 features with p < 0.05: age, performance status, stem cell transplantation, PLT, PDW, MPV, PLCR, K, AST, LDH, BUN, creatinine, and CRP (Table 3). Meanwhile, univariate Cox regression analyses to determine prognostic factors associated with progression-free survival revealed 11 features with p < 0.05: age, stem cell transplantation, RBC, HCT, RDW, Na, LDH, albumin, globulin, albumin/globulin (AG) ratio, and CRP (Table 4). The important prognostic factors determined by multivariate Cox regression analyses associated with overall survival were two features: AST and LDH (Table 3). The prognosis of patients with high levels of AST or LDH was worse. The optimal cut-off points according to these parameters were not determined, because the investigated patient numbers in the present study were small. Therefore, further studies are needed to clarify the optional cut-off points. Meanwhile, the important prognostic factors determined by multivariate Cox regression analyses associated with progression-free survival were not detected.

**Table 3:** Results of univariate and multivariate Cox regression analyses for overall survival (p < 0.05)

**Features**	**Hazard**	**95% CI**	**p value**
	**ratio**	**for hazard ratio**	
*Univariate Cox regression*			
Age	1.09	1.01-1.17	0.027
Performance status	1.82	1.01-3.25	0.045
Stem cell transplantation	0.25	0.07-0.89	0.033
PLT	0.91	0.83-0.99	0.033
PDW	1.26	1.02-1.56	0.031
MPV	1.87	1.16-3.02	0.010
PLCR	1.08	1.02-1.15	0.013
K	2.15	1.09-4.27	0.028
AST	1.02	1.00-1.04^1^	0.017
LDH	1.01	1.00-1.01^2^	0.005
BUN	1.05	1.03-1.08	0.000
Creatinine	1.31	1.02-1.69	0.032
CRP	1.28	1.06-1.55	0.009
*Multivariate Cox regression*			
AST	10.6	1.01-112	0.049
LDH	1.17	1.01-1.36	0.039

^1^95% CI for hazard ration of AST is exactly 1.004-1.043.

^2^95% CI for hazard ration of LDH is exactly 1.002-1.010.

**Table 4:** Results of univariate Cox regression analyses for progression free survival (p < 0.05)

**Features**	**Hazard**	**95% CI**	**p value**
	**ratio**	**for hazard ratio**	
*Univariate Cox regression*			
Age	1.06	1.01-1.11	0.026
Stem cell transplantation	0.42	0.19-0.97	0.042
RBC	0.99	0.98-1.00	0.014
HCT	0.87	0.78-0.97	0.013
RDW	1.07	1.02-1.13	0.010
Na	0.88	0.80-0.96	0.003
LDH	1.01	1.00-1.01	0.000
Albumin	0.40	0.17-0.94	0.035
Globulin	1.27	1.03-1.57	0.028
AG ratio	0.35	0.14-0.83	0.018
CRP	1.20	1.06-1.36	0.005

Hepatic dysfunction was observed in 7 patients (15.9%). These patients were serologically negative for hepatitis B and C. Also, abdominal ultrasonography or computerized tomography demonstrated that it was related to liver involvement with MM (2 cases: 4.5%), fatty liver (2 cases: 4.5%), gallstone (1 cases: 2.3%), and postcholecystectomy (2 cases: 4.5%), respectively.

### Safety

All 44 patients were evaluated for toxicity using the Common Terminology Criteria for Adverse Events (CTCAE) version 4.0. Hematologic toxicity was reversible and manageable. Patients reported grade 3/4 anemia (13.6%), grade 3/4 neutropenia (15.9%), and grade 3/4 thrombocytopenia (22.7%) (Table 5). Although grade 4 neutropenia occurred, the patients were treated with granulocyte colony-stimulating factors. Patients with grade 3/4 anemia or grade 4 thrombocytopenia had blood or platelet transfusions. The most common grade 3/4 nonhematologic toxicities were tumor lysis syndrome (6.8%). No treatment-related deaths were noted. Interstitial pneumonitis, ileus, herpes zoster infections, peripheral neuropathy, and fever were also observed. Because these toxicities were mild, bortezomib dose omission or reduction were rare.

**Table 5:** All grade 3 and 4 adverse events (n = 44)

**Adverse event**	**Grade 3**	**Grade 4**		
	N	%	N	%
Anemia	5	11.4	1	2.3
Neutropenia	2	4.5	5	11.4
Thrombocytopenia	4	9.1	6	13.6
Tumor lysis syndrome	2	4.5	1	2.3
Interstitial pneumonitis	1	2.3	0	0
Ileus	1	2.3	0	0
Herpes zoster infections	1	2.3	0	0

## Discussion

Multiple myeloma accounts for 10% of all hematologic cancers [9]. With conventional treatments, MM remains an essentially incurable disease with a median survival of 3–4 years [10]. Treatment of MM remains highly individualized, with multiple factors that play a role in determining the best course of therapy. Patient-specific criteria such as age of onset, whether the patient is symptomatic at the time of diagnosis, and any detected high-risk cytogenic abnormalities are all considerations when selecting a regimen. Bortezomib has been approved by the Swiss Agency for Therapeutic Products (Swissmedic, https://www.swissmedic.ch/) for the treatment of MM in the frontline setting in combination with MP and in patients with relapsed/refractory MM who have received at least one prior therapy [11]. Bortezomib offers a novel approach to the treatment of MM in Phase 2 or 3 clinical trials producing rapid control [12-14]. The achievement of a complete or partial response to bortezomib as a salvage treatment is associated with a significantly longer survival [12]. Several studies of single-agent bortezomib at doses of 1.3 mg/m^2^ as first-line, or second-line or latter, therapy have median time to progression ranging from 1.4 to 17.3 months, median progression-free survival time ranging from 5.0 to 17.0 months, and median overall survival time from 14.6 to 29.8 months, in MM [12-22] (Table 6). Our median time to progression of 14.9 months, median progression-free survival time of 14.9 months, and median overall survival time survival time of 38.3 months, in patients treated with MM was also comparable to other trials of single-agent therapy.

**Table 6:** Activity of bortezomib in multiple myeloma

**Study**	**Bortezomib dose regimen**	**In combination with dexamethasone**	**As the *n*** **th chemotherapy**	**Assessable patients**	**TTP months**	**PFS months**	**OS months**
**Retrospective study**							
Min et al. , 2007	1.3 mg/m^2^ twice	Yes	2-4	21	12.1	NR	NR
[15]	weekly for 2						
	weeks in a 21-day						
	cycle						
Corso A, et al.	1.3 mg/m^2^ on days	Yes	≥ 2	61	5.6	5.4	14.6
2009	1, 4, 8, and 11 of a						
[16]	21-day cycle						
Ohguchi H, et al.	1.3 mg/m^2^on days	Yes	≥ 2	40	8.7	NR	NR
2009	1, 4, 8, and 11 of a						
[17]	21-day cycle						
Present study	1.3 mg/m^2^ on days	Yes	1-9	44	14.9	14.9	38.3
	1, 4, 8, and 11 of a						
	21-day cycle						
	or						
	1.3 mg/m^2^ intravenously						
	1, 8, 15, and 22 of every						
	35-day cycle						
**Phase II**							
Richardson et al. 2003	1.3 mg/m^2^ on days	Yes	≥ 2	202	7.0	NR	16.0
[12]	1, 4, 8, and 11 of a						
	21-day cycle						
Jagannath et al. 2004	1.3 mg/m^2^twice	Yes	2-8	26	11.0	NR	NR
[13]	for 2 weeks in a						
	21-day						
Richardson PG, et al.	1.3 mg/m^2^ on days	No	1	64	17.3	17.0	NR
2009	1, 4, 8, and 11 of a						
[18]	21-day cycle						
**Phase III**							
Kane et al. 2006	1.3 mg/m^2^ on days	Yes	≥ 2	333	6.2	5.7	NR
[19]	1, 4, 8, and 11 of a						
	21-day cycle						
	(4 doses) for up to						
	8 cycles, followed by						
	up to 3 additional						
	5-week cycles of once						
	weekly dosing						
	(4 doses)						
Orlowskiet al. 2007	1.3 mg/m2 on days	No	≥ 2	322	6.5	6.5	NR
[20]	1, 4, 8, and 11 of a						
	21-day cycle						
Richardson et al., 2007	1.3 mg/m2 on days	No	2-4	333	1.4	NR	29.8
[21]	1, 4, 8, and 11						
	for eight 3-week						
	cycles, then on days						
	1, 8, 15, and 22 for						
	three 5-week						
	maintenance						
	cycles						
Sonneveld P, et al.	1.3 mg/m2 on days	No	≥ 2	184	6.8	NR	NR
2008	1, 4, 8, and 11 of a						
[22]	21-day cycle						

Figure in parentheses indicate percentages. NR = Not reported; OS = overall survival; PFS = progression-free survival; TTP: time to progression.

In this study, the factors significantly associated with overall survival were AST and LDH levels in patients with bortezomib. The present study also suggests the possibility that the prognosis of patients with high levels of AST and LDH might be worse than that of patients with low levels of these parameters. The blood test for AST is usually used to detect liver damage. A review of 869 cases of multiple myeloma seen at the Mayo Clinic from 1960 through 1971 revealed that initial findings was a palpable liver in 21% [23]. It was reported that abnormalities in liver function were characteristic, and out of 37 cases of MM, serum level of AST was increased in 22 (59.5%) [24]. In the present study, as mentioned above, hepatic dysfunction was observed in 7 patients (15.9%). These patients were serologically negative for hepatitis B and C. Also, abdominal ultrasonography or computerized tomography demonstrated that it was related to liver involvement with MM, fatty liver, gallstone, and postcholecystectomy. Therefore, there is a possibility that the prognosis of patients with hepatic dysfunction might be worse than that of patients without this. Moreover, Walz-Mattmüller, et al. [25] previously investigated the incidence and pattern of liver involvement in liver specimens from 25 cases of MM histologically and immunohistochemically. Liver infiltration was found in 32% of MM specimens. Moreover, diffuse, non-destructive infiltration was most common, and the infiltration was mainly sinusoidal, and also, nodular infiltration was seen. Furthermore, Oshima et al. [26] reported that hepatic invasion was observed in 15 patients (28.8%) in 52 consecutively autopsied cases with MM, but among them, diffuse tumor involvement was seen only on macroscopic examination in 8 patents (15.4%), and liver infiltration by MM was frequent in patients with IgA-type myeloma. Consistent with this, further investigation are needed to clarify the mechanism of liver damage in MM patients, because AST was one of the important prognostic factors determined by multivariate Cox regression analyses associated with overall survival, although in the present study, we did not obtain liver specimens for all patients with liver dysfunction. On the other hand, it was reported that high serum LDH is associated with features of advanced disease and inferior survival in multiple myeloma [27]. Therefore, we speculate that the worse prognosis of patients with high levels of AST and LDH might be associated with the advanced stages of diseases of these MM patients.

Greipp et al. previously reported the association between higher Durie-Salmon stage or ISS stage and worse outcome [7]. However, in the present study, our data did not reveal a significant impact for a Durie-Salmon stage or ISS stage. Staging a patient under the Durie-Salmon-system requires results from a bone marrow biopsy, bone survey, serum electrophoresis, and values for haemoglobin, haematocrit and serum calcium, and meanwhile, the ISS-stage utilizes a combination of serum β2 microglobulin and serum albumin. Therefore, we speculated that the association between a higher Durie-Salmon stage or ISS stage and worse outcome was not observed, because Durie-Salmon stage or ISS stage did not correlate with high AST and LDH levels. Also, consistent with this, several investigators reported the prognostic value of LDH in MM patients [28,29], This, however, was not incorporated in any widely used staging system, although its has an ability to identify patients with an especially adverse outcome [30,31]. Because the investigated patient numbers in the present study were small, further investigations are also needed to clarify this matter.

According to the issue that a high AST and LDH were not associated with progression-free survival, since the late 70s, the relationship between hematological malignancies and elevated LDH has been intensively studied [32]. In aggressive lymphoma patients, increased LDH was found linked to high tumor burden and turnover [33]. In patients that received autologous stem-cell transplantation for multiple myeloma, a high LDH was an independent prognostic factor for both overall survival and progression-free survival [34]. However, in the present study, although LDH and AST had an independent prognostic value for overall survival, these were not statistically significant indicators for progression-free survival. This may be a reflection of inadequate sample size.

The toxicity profile in our study was generally acceptable (Table 5). The major toxicity was myelosuppression; the incidence of grade 3/4 toxicity was 22.7% for thrombocytopenia, 15.9% for neutropenia, 6.8% for anemia, and 6.8% for tumor lysis syndrome. Although grade 4 neutropenia occurred, the patients were treated with granulocyte colony-stimulating factors. Patients with grade 3/4 anemia or grade 4 thrombocytopenia had blood or platelet transfusions. Treatment-related deaths were not observed. Consistent with this, the first trial was reported by Richardson et al. [12], who treated 193 patients with MM with bortezomib. Grade 3 adverse events included thrombocytopenia (in 28% of patients), fatigue (in 12%), peripheral neuropathy (in 12%), and neutropenia (in 11%), meanwhile, grade 4 events (thrombocytopenia, neutropenia, vomiting, diarrhea, weakness) occurred in 14 percent of patients; otherwise, no severe adverse events occurred.

In conclusion, recent clinical studies, including this study, demonstrate that bortezomib has a therapeutic effect on MM. This study also suggests that bortezomib and dexamethasone are well tolerated in the treatment of MM. In the present study, we have documented the strengths of the study that there is a possibility that the prognosis of patients with high levels of AST and LDH might be worse than the prognosis of patients with low levels of AST and LDH. According to the weakness of this study, although AST and LDH had independent prognostic value for overall survival, we did not demonstrate that these were statistically significant indicators for progression-free survival. This may be a reflection of inadequate sample size. The presented study is a retrospective study, and therefore, these results should be confirmed in further prospective studies.

## Abbreviations

CED: Cyclophosphamide, etoposide, dexamethasone; CP: Cyclophosphamide, prednisolone; CAD: Cyclophosphamide, adriamycin, dexamethasone; CR: Complete response; DEX pulse: Dexamethasone pulse therapy; EPOCH: Etoposide, prednisone, vincristine, cyclophosphamide, doxorubicin; MD: Melphalan, dexamethasone; MCNU-VMP: MCNU, vindesine, melphalan, prednisolone; MM: Multiple myeloma; MP: Melphalan, prednisolone; MPT: Melphalan prednisone thalidomide; HDD: High dose dexamethasone; INF α: Interferon alpha; PD: Progressive disease; RD: Lenalidomide, dexamethasone; ROAD: MCNU, vincristine, melphalan, dexamethasone; VAD: Vincristine, adriamycin, dexamethasone.

## Competing interests

All authors declare that they have no competing interests.

## Authors’ contribution

TK conception and design, acquisition, analysis and interpretation of data, drafting the manuscript, revising the manuscript, final approval of the version to be published. TI acquisition of data, manuscript revision. TN, YO, MK, AK, KK, FM and HN acquisition of data. ST and MA acquisition, analysis and interpretation of data. YS analysis of data. All authors read and approved the final manuscript.

## Pre-publication history

The pre-publication history for this paper can be accessed here:

http://www.biomedcentral.com/1471-2407/14/462/prepub
